# Tristetraprolin functions in cytoskeletal organization during mouse oocyte maturation

**DOI:** 10.18632/oncotarget.10755

**Published:** 2016-07-21

**Authors:** Xiaohui Liu, Xiaoyan Li, Rujun Ma, Bo Xiong, Shao-Chen Sun, Honglin Liu, Ling Gu

**Affiliations:** ^1^ College of Animal Science and Technology, Nanjing Agricultural University, Nanjing, China; ^2^ Center of Reproductive Medicine, Jinling Hospital, Nanjing, China

**Keywords:** oocyte, meiosis, cytoskeleton, reproduction, TTP

## Abstract

Tristetraprolin (TTP), a member of TIS11 family containing CCCH tandem zinc finger, is one of the best characterized RNA-binding proteins. However, to date, the role of TTP in mammalian oocytes remains completely unknown. In the present study, we report the altered maturational progression and cytokinesis, upon specific knockdown of TTP in mouse oocytes. Furthermore, by confocal scanning, we observe the failure to form cortical actin cap during meiosis of TTP-depleted oocytes. Loss of TTP in oocytes also results in disruption of meiotic spindle morphology and chromosome alignment. In support of these findings, incidence of aneuploidy is accordingly increased when TTP is abated in oocytes. Our results suggest that TTP as a novel cytoskeletal regulator is required for spindle morphology/chromosome alignment and actin polymerization in oocytes.

## INTRODUCTION

Tristetraprolin (TTP, ZFP36) is the founding member of tandem CCCH zinc fingers containing proteins that negatively regulates RNA stability, promotes RNA degradation. It binds directly to adenylate-uridylate-rich elements (AREs) located in the 3′ UTR of target mRNAs [1]. AREs are the important elements regulating post-transcription, and have been detected in around 10% of the human transcriptomes [2]. Since its discovery as a regulator of TNFα mRNA stability nearly two decades ago, TTP has become one of the best understood post-transcriptional regulators associated with tumors and inflammation [3, 4]. It has been showed that TTP regulates the expression of MyoD, CCL3 and IL-23 in an mRNA-decay dependent way [5–8]. Increased systemic levels of TTP, secondary to increased stability of its mRNA throughout the body, can protect mice against immune-mediated inflammatory pathologies [9]. In human glioma cells, TTP induced by resveratrol suppresses cell growth and results in cell apoptosis [10]. TTP can also affect the balance of anabolic and catabolic gene expression in human chondrocytes [11]. It is worth noting that TTP is expressed in growing mouse oocytes [12], but its role in germ cells remains completely unknown.

In mammalian females, oocytes are arrested at prophase I after homologous recombination during development of fetus. Under the stimulation of luteinising hormone (LH) surge, oocytes reinitiate meiosis, proceed through meiosis I (MI) division, followed by the second arrest at metaphase II (MII) awaiting for fertilization [13]. This process is also called oocyte meiotic maturation. Meiotic progression requires exquisitely coordinated maturation of both nucleus and cytoplasm, which including multiple physiological events, such as germinal vesicle breakdown (GVBD), spindle formation, chromosome movement and polar body extrusion [14]. The redistribution of organelles, microtubules, actin filaments, and other cytoskeleton-related proteins provides the framework for these diverse cellular processes. The critical involvement of actin in cortical polarization and asymmetric spindle positioning during oocyte meiotic division has been observed in a wide range of mammalian species. Particularly, the establishment of actin cap is one of the predominant features of oocyte polarity [15]. Any errors in chromosome separation during meiosis could lead to aneuploidy in oocytes, which are considered the major genetic causes responsible for miscarriage and infertility [13]. Although numerous molecules have been reported to be involved in oocyte maturation, pathways and mechanisms that modulate this process remain to be discovered.

The goal of this study was to assess the function of TTP in mouse oocytes. By knocking down TTP with specific small interfering RNA (siRNA), our data showed that TTP is involved in modulating meiotic maturation, particularly the cytoskeletal organization and cell division.

## RESULTS

### Cellular localization of TTP during oocyte maturation

**Figure 1:**
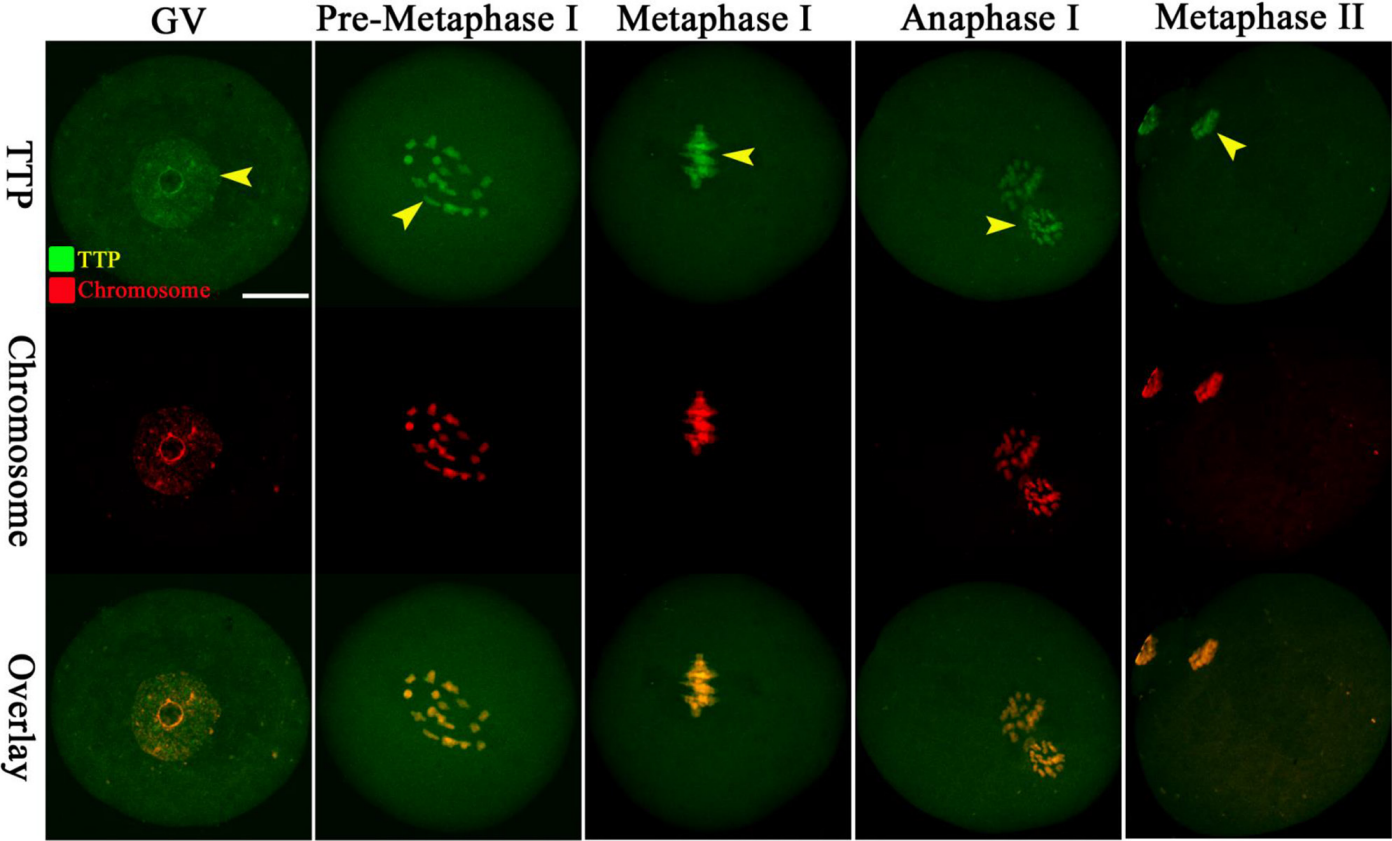
Cellular localization of TTP in mouse oocytes Oocytes at GV, Pre-Metaphase I, Metaphase I, Anaphase I and Metaphase II stages were immunolabeled with TTP antibody (green) and counterstained with propidium iodide for DNA (red), respectively. Representative confocal images are shown. TTP signals are indicated by arrowheads. 30 oocytes were examined for each group. Scale bar, 25 μm.

By immunofluorescent labeling coupled with confocal microscopy, we examined TTP distribution during mouse oocyte maturation. Immunostaining clearly showed the presence of TTP in oocytes at different developmental stages (Figure 1, green). At GV stage, TTP fluorescence signals predominantly accumulate in the nucleus. It is worth noting that, accompany with the resumption of meiosis, TTP appears to be largely colocalized with the chromosomes from pre-metaphase I to metaphase II stages. Such a specific distribution pattern indicates that TTP may be involved in regulation of chromosome-related oocyte events.

### TTP knockdown affects maturational progression of oocytes

**Figure 2:**
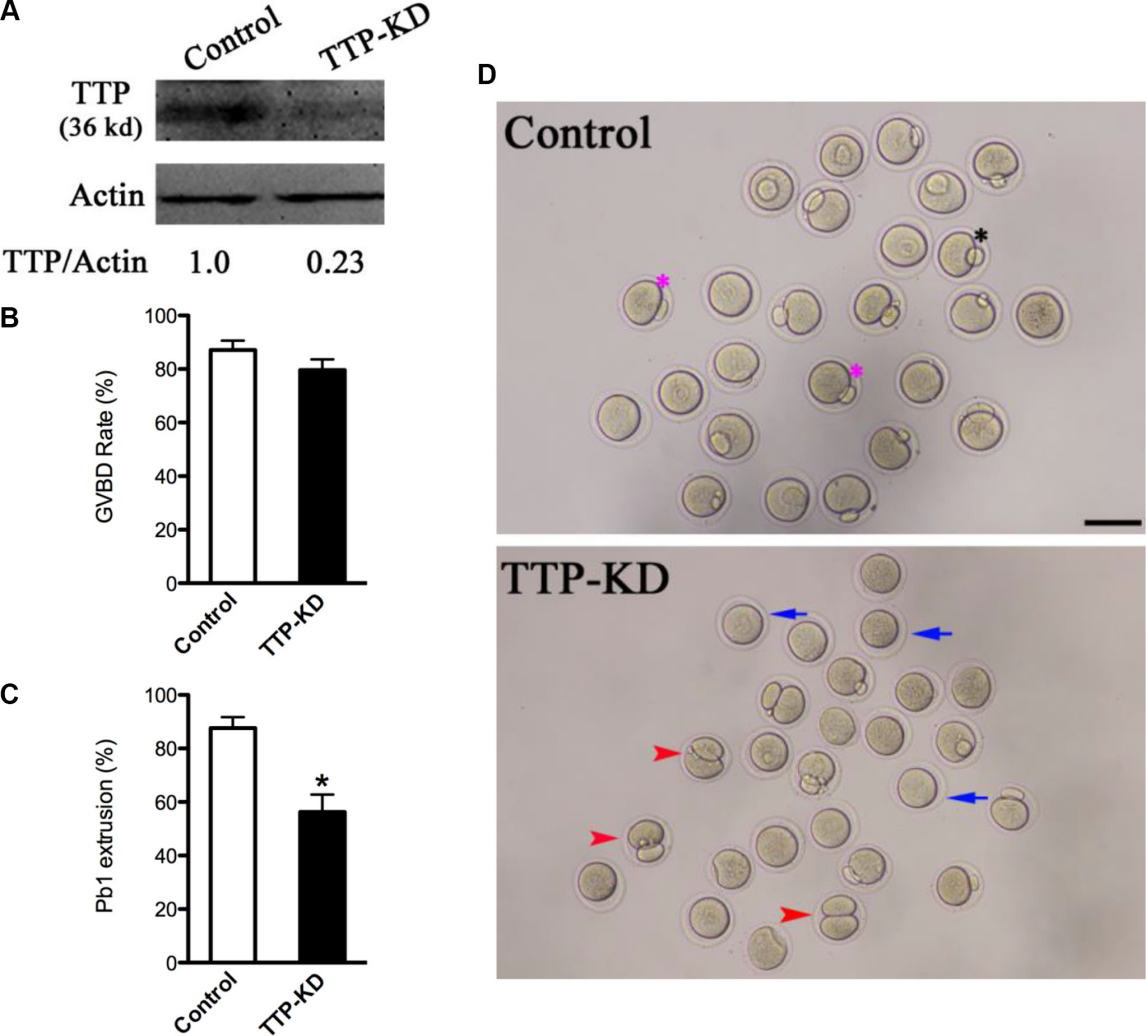
Effects of TTP knockdown on oocyte maturation Fully-grown oocytes injected with TTP-siRNA were arrested at GV stage with milrinone for 20 hours, and then cultured in milrinone-free medium to evaluate the maturational progression. Negative control siRNA was injected as control. (**A**) Knockdown of endogenous TTP protein expression after TTP-siRNA injection was verified by Western blot analysis with actin as a loading control. Band intensity was measured by Image J software, and the ratio of TTP/actin expression was normalized. (**B** and **C**) The rate of GVBD and Pb1 extrusion in control (*n* = 162) and TTP-KD (*n* = 138) oocytes. Data were expressed as mean ± SD from three independent experiments. *p < 0.05 vs control. (**D**) Phase-contrast images of control siRNA and TTP-siRNA injected oocytes. Pink asterisks indicate the normal matured oocytes with first polar body; blue arrows indicate the oocytes that fail to extrude polar bodies; red arrowheads denote oocytes with apparent symmetrical division. Scale bar, 80 μm.

Given the distribution pattern of TTP, we tried to explore its function during oocyte maturation. We microinjected *TTP*-specific siRNA into fully-grown oocytes, control siRNA was injected as negative control. Western blotting verified that TTP protein level was remarkably knocked down (Figure 2A). Then, we analyzed how the loss of TTP affected oocyte maturation. Oocyte maturation progression includes meiotic resumption indicated by GVBD, microtubules organizing into the metaphase I (MI) spindle, chromosomes condensation and aligning at MI metaphase plate, execution of the MI division, extruding the first polar body (Pb1), and then proceeding to metaphase II (MII ) until fertilization. Our results showed that TTP depletion had little effect on the meiotic resumption, evidenced by the rate of GVBD (79.6 ± 4.0% vs. 87.1 ± 3.5% control, *p* > 0.05; Figure 2B). However, the proportion of Pb1 extrusion was decreased in TTP-KD oocytes compared with control ones (56.3 ± 6.5 vs. 87.6 ± 4.1% control, *p* < 0.05; Figure 2C), indicative of the involvement of TTP in the meiotic process. After 14 hours culture, most control oocytes completed meiosis I and formed Pb1 (Figure 2D, pink asterisks). Notably, a high frequency of TTP-KD oocytes were unable to complete meiosis showing no polar bodies (Figure 2D, blue arrowheads), or experienced symmetric division showing 2-cell like phenotype (Figure 2D, red arrowheads). Altogether, these observations suggest that TTP is essential for oocyte maturation and meiotic division.

### TTP knockdown results in the failure to form actin cap in oocytes

**Figure 3:**
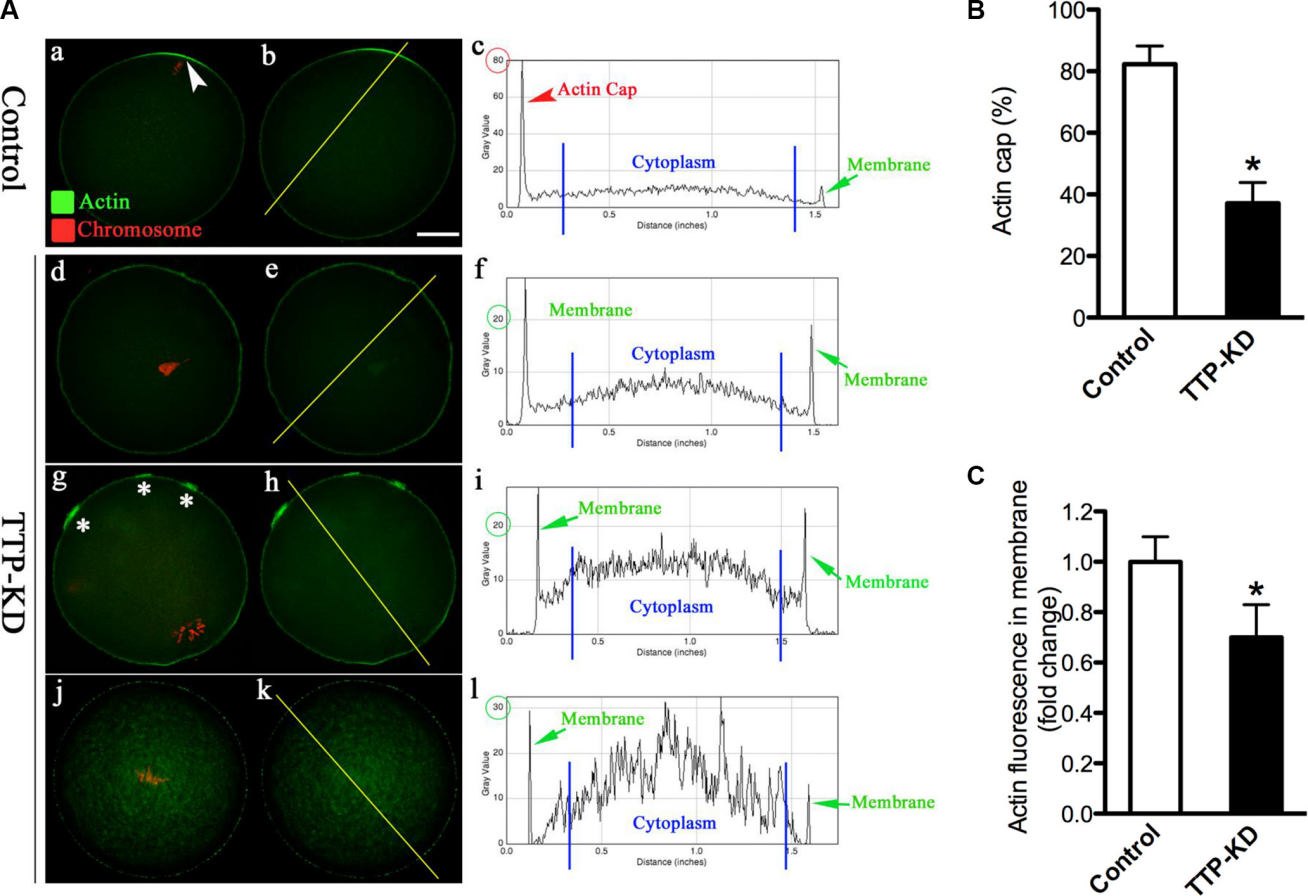
TTP knockdown disrupts the formation of actin cap during oocyte maturation MII oocytes were labeled with phalloidin to visualize actin (green), counterstained with propidium iodide for chromosomes (red), and then were imaged for fluorescence quantification. (**A**) Representative images show the actin distribution in control and TTP-KD oocytes. Arrowhead indicates the position of actin cap. Right graphs are fluorescence intensity profiles of phalloidin in oocytes. Lines were drawn through the oocytes, and pixel intensities were quantified along the lines. (**B**) Quantitative analysis of the proportion of actin cap formation in control and TTP-KD oocytes. (**C**) Quantification of the mean fluorescence intensity of phalloidin in membrane of oocytes. At least 50 oocytes were examined for each group, and data were expressed as the mean ± SD from three independent experiments. Scale bar: 25 μm. *p < 0.05 vs controls.

Mammalian oocyte maturation is a complex process that involves extensive rearrangements of actin filaments and microtubules [16]. It has been well established that oocytes require actin to maintain their shape, for growth, polarization and replication [17]. Actin cap formation is one of the predominant features of oocyte polarization. To examine the effect of TTP on actin polymerization in more details, matured TTP-KD and control oocytes were labeled with actin tracker phalloidin, counterstained with propidium iodide for chromosomes, and then quantitative analysis was performed. As shown in Figure 3Aa, actin caps were clearly observed on membrane of normal MII oocytes (arrowhead), evidenced by the fluorescence plot profiling (Figure 3Ab–c). By contrast, failure to form actin cap was readily detected when TTP was abated in mouse oocytes (Figure 3A). Several major phenotypes were observed, including the lack of actin cap (Figure 3Ad–f), multiple micro-caps of actin (Figure 3Ag–i), and elevated actin intensity in the cytoplasm (Figure 3Aj–l). Moreover, quantitative analysis demonstrated that both actin cap formation and fluorescence intensity on cortex were significantly reduced in TTP-depleted oocytes in comparison to controls (Figure 3B and 3C). These results indicate that loss of TTP disrupted the microfilament polymerization and actin cap formation, which may contribute to the meiotic division defects we mentioned above.

### Proper spindle/chromosome organization in mouse oocyte depends on TTP

**Figure 4:**
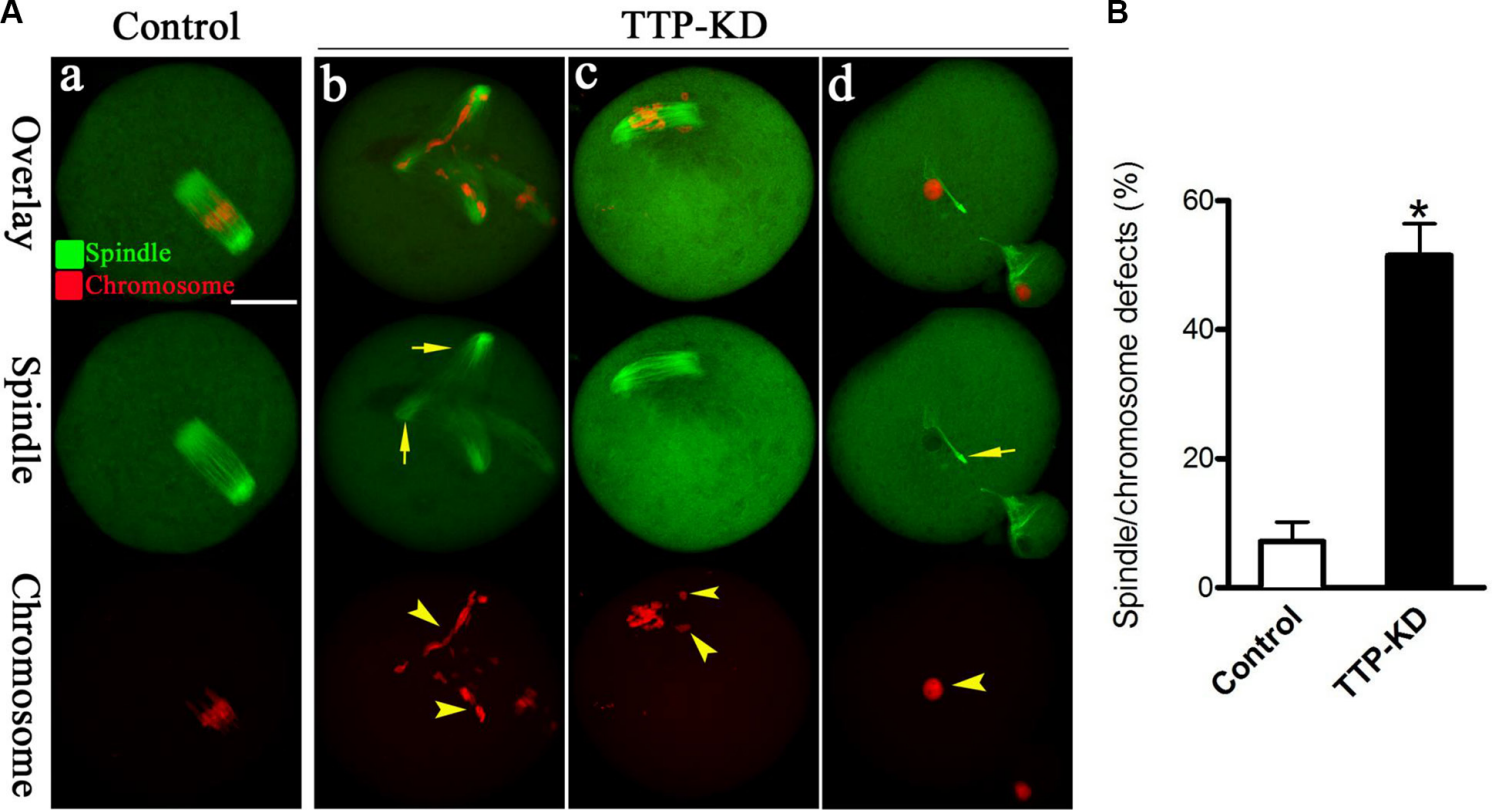
Effects of TTP knockdown on spindle organization and chromosome alignment in oocyte meiosis (**A**) Oocytes at metaphase I stage were stained with anti-tubulin antibody to visualize spindle (green) and counterstained with propidium iodide to visualize chromosomes (red). Representative confocal images from control and TTP-KD oocytes are shown. Normal oocytes (a) present a bipolar barrel-shaped spindle and well-aligned chromosomes on the metaphase equator. Spindle defects (arrows) and chromosomes misalignment (arrowheads) were frequently observed in TTP-KD oocytes (b–d). (**B**) Quantification of control (*n* = 118) and TTP-KD (*n* = 127) oocytes with abnormal spindles/chromosomes. Data were expressed as mean ± SD from 3 independent experiments in which at least 100 oocytes were analyzed. *p < 0.05 vs. controls. Scale bar, 25 μm.

The specific positioning of TTP on chromosome and its effects on maturation progression prompted us to hypothesize that TTP might play a regulatory role in the assembly of meiotic apparatus. For this purpose, mouse oocytes from control and TTP-KD groups were immunolabeled with anti-tubulin antibody to visualize the spindle and counterstained with propidium iodide for chromosomes. As shown in Figure 4Aa, confocal microscopy and quantitative analysis revealed that most control oocytes at metaphase stage showed a typical barrel-shaped spindle and well-organized chromosomes at the equator plate. In contrast, a high frequency of chromosome misalignment and severe spindle morphology defects (51.5 ± 4.9 vs. 7.2 ± 3.0% control, *p* < 0.05; Figure 4B) were observed in TTP-KD oocytes, displaying multipolar spindles (Figure 4Ab, arrows), collapsed spindles (Figure 4Ad, arrow), and displacement of several chromosomes from equator (Figure 4Ac, arrowheads). These findings suggest that, in many cases, TTP-depleted oocytes cannot properly organize the meiotic spindle and align the meiotic chromosomes.

### Incidence of aneuploidy is increased in TTP-depleted eggs

**Figure 5:**
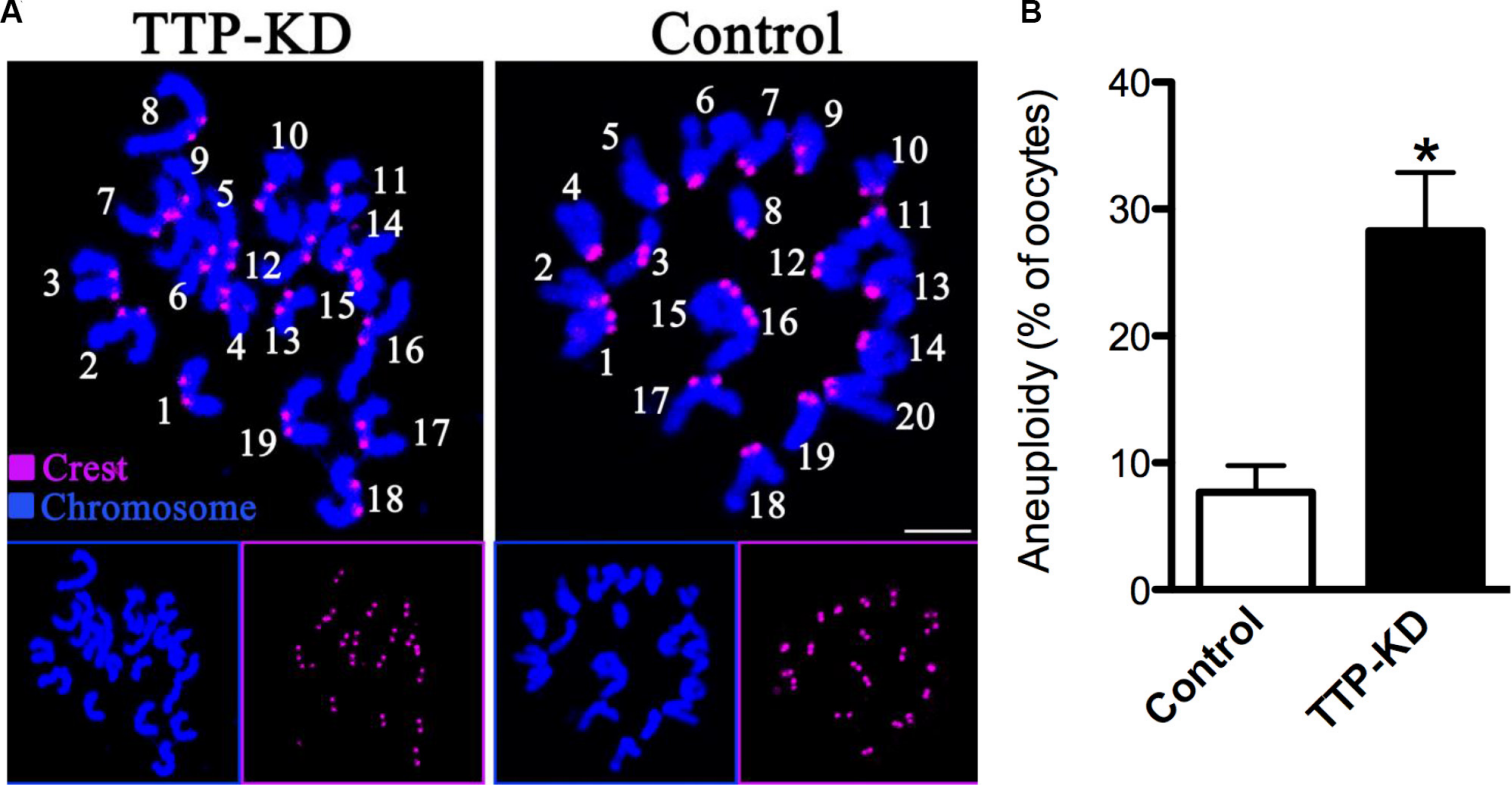
Increased aneuploidy in oocytes loss of TTP (**A**) Chromosome spread and kinetochore labeling of control and TTP-KD oocytes at MII stage. Representative confocal images of euploid and aneuploid oocytes are shown. (**B**) Quantification of aneuploidy in control and TTP-KD oocytes. Data are expressed as mean ± SD percentage, and 30 oocytes per group were analyzed. *P < 0.05 vs. controls.

Given the fact that TTP knockdown led to high frequency of spindle defects and chromosome misalignment, we further analyzed the karyotype of MII stage oocytes by chromosome spreading and kinetochore labeling to see whether oocytes deficient of TTP would act to generate aneuploidy eggs. As shown in Figure 5A (representative images of euploidy and aneuploidy), we found that the proportion of aneuploid eggs in TTP-depleted group is about 4-fold increase compared to control group (28.3 ± 4.6 vs. 7.7 ± 2.1% control, *P* < 0.05; Figure 5B). Taking together, these data suggested that oocytes depleted of TTP were prone to generate aneuploid eggs, probably due to the defective assembly of meiotic structures.

## DISCUSSION

Mammalian oocytes undergo induced arrest at the diplotene stage of prophase I during meiosis in the ovary. Upon hormonal surge, oocytes will exit the prophase I arrest and resume meiosis. All stages from meiotic resumption, starting with germinal vesical breakdown until the next arrest where oocytes are fertilized, belong to the maturation process [18]. And this process includes bipolar barrel-shaped spindle organization, chromosome alignment, polarization establishment, and polar body extrusion [19]. Errors at any step in this process can lead to abnormal chromosome segregation and the resultant aneuploid eggs. In this study, by immunofluorescence labeling and confocal microscopy, we found that specific knockdown of TTP in mouse oocytes resulted in remarkable defects of spindle morphology and chromosome movement (Figure 4). In consistent with this, the aneuploidy of TTP-KD oocytes was much higher than that of control oocytes (Figure 5). Interestingly, mutation of another member of TIS11 family, ZFPL2, caused failure of both ovulation and oocyte maturation *ex vivo* in mouse [20]. Taking these observations together, we conclude that TTP is required for orderly meiosis of mammalian oocytes and promotes the production of high quality eggs.

Oocyte maturation is characterized by the succession of two asymmetric divisions each producing a small polar body and a big functional haploid egg [21]. The asymmetric divisions are dictated by that the radially symmetric oocyte is transformed into a highly polarized MII-arrested egg. Actin filaments, one of the main cytoskeletal systems, are involved in the regulation of these dynamic events, like actin cap formation and cortical granual free domain [17, 22]. The reduced ability of oocytes to polymerize F-actin is a major reason for the failure of polar body extrusion during maturation [23]. It has been reported that meiosis I chromosome movement was biphasic, one was driven by consecutive actin-based pushing forces through Fmn2 and Arp2/3 complex, the second was driven via actin-mediated cytoplasmic streaming [24]. Here we identified that, in addition to the disrupted actin cap (Figure 3), oocytes depleted of TTP presented other phenotypes including failure to extrude polar body (Figure 2), spindle disorganization, and chromosome misalignment (Figure 4). Collectively, on the basis of these data, we propose that knockdown of TTP induces defective actin polymerization, which in turn perhaps impairs microtubule stability and chromosome movement during oocyte meiosis, resulting in the generation of aneuploid eggs.

TTP is the founding member of tandem CCCH zinc fingers containing proteins. As a member of TIS11 family, TTP has been demonstrated to be able to bind to AREs in the 3′UTRs of specific mRNAs and modulates their stabilities [9]. Similarly, *t*wo zinc finger proteins OMA-1 and OMA-2 in *C. elegans* were found to regulate oocyte meiotic maturation via MAP kinase and MYT-1 activities [25]. Loss of Gla-3, a TIS11-like zinc-finger containing protein, also results in increased germline apoptosis and reduced brood size due to defective pachytene exit from meiosis I through the MAPK signaling cascade [26]. Notably, cAMP/PKA pathway was suggested to be involved in *ZFP36L2* mutation associated meiotic arrest [20]. Furthermore, sperm chromatin-induced cortical reorganization can be blocked by the inhibitors of microfilament assembly or disassembly, while active MAPK is required for this reorganization [27]. Generally, the actions of gonadotrophins, FSH and LH, on oocyte meiotic resumption are believed to be mediated in large part through increasing the production of cAMP and subsequent activation of MAPK [28]. Here we show that TTP is co-localized with meiotic chromosomes (Figure 1) and participates in the control of oocyte meiosis (Figure 2). Thus, these findings lead to a hypothesis that MAP kinase may be an important target pathway mediating the effects of TTP on mammalian oocytes. Additional experiments will be required to understand the potential mechanisms.

Failure to establish the asymmetry of meiotic structure position significantly affects oocytes polarization and polar body emission, which finally contributes to the decreased reproductive potential in females [29]. In the present study, we reveal that TTP as a cytoskeletal regulator that is required for spindle morphology/chromosome alignment and actin polymerization in oocytes. Our findings open a new area for understanding mechanisms controlling oocyte meioiss as well as assessing oocyte quality probably with the critical zinc finger proteins.

## MATERIALS AND METHODS

All chemicals and culture media were purchased from Sigma (St. Louis, MO, USA) unless stated otherwise. ICR mice were used in this study. All experiments were approved by the Animal Care and Use Committee of Nanjing Agricultural University and were performed in accordance with institutional guidelines.

### Oocyte collection and culture

Approximately 46–48 h after injection of 5 IU Pregnant Mares Serum Gonadotropin (PMSG), fully-grown immature oocytes were harvested from ovaries of 6–8 week old female ICR mice. Enclosed cumulus cells were removed by repeatedly pipetting, and then oocytes were cultured in M16 medium under mineral oil at 37°C in a 5% CO_2_ incubator. At appropriate time points (0 h, 3 h, 7 h, 9 h, 14 h), oocytes were selected for the following assays. Phase contrast microscope was used to observe germinal vesicle (GV) breakdown and polar body (Pb) extrusion to evaluate the meiotic progression of mouse oocytes.

### siRNA knockdown

Microinjection of siRNA into the cytoplasm of fully-grown immature oocytes was used to knock down TTP. siRNA (GenePharma, Shanghai, China) was diluted with water to give a stock concentration of 1 mM, and then 2.5 picoliter solution was injected into oocytes with a Narishige microinjector. A negative control siRNA was set. After injections, oocytes were arrested at GV stage with 2.5 μM milrinone for 20 hours, and then were cultured in milrinone-free M2 medium for maturation.

TTP-siRNA:

5′- GGACUUUGGAACAUAAACUTT-3′;

5′-AGUUUAUGUUCCAAAGUCCTT-3′

Negative control siRNA:

5′-UUCUCCGAACGUGUCACGUTT-3′;

5′-ACGUGACACGUUCGGAGAATT-3′.

### Western blotting

Approximately 100 oocytes was lysed in Laemmli sample buffer with protease inhibitor, and heated at 100°C for 5 min. Proteins were separated on 10% SDS-PAGE, transferred to PVDF membranes, blocked in TBST (TBS containing 0.1% Tween 20) with 5% nonfat milk at room temperature for 1 hour. Then the PVDF membrane was incubated overnight at 4°C with 1:1000 dilution of rabbit anti-TTP antibody (Millipore, Cat# ABE285) or 1:5000 dilution of anti-Actin antibody (Sigma, Cat# A5441 ). After 3 times wash in TBST, membranes were incubated with proper HRP-conjugated secondary antibodies for 1 hour at room temperature, and then processed using an ECL Plus Western Blotting Detection System. Each experiment was repeated at least three times.

### Immunofluorescence and confocal microscopy

For immunostaining of TTP and actin, oocytes were fixed in 4% paraformaldehyde in PBS (pH 7.4) for 30 minutes and permeabilized in 0.5% Triton-X 100 for 20 min at room temperature. Then oocytes were blocked with 1% BSA-supplemented PBS for 1 h and incubated at 4°C overnight with rabbit anti-TTP antibody (1:100, Millipore, MA), Phalloidin-FITC (1:100, Sigma, MO) or mouse monoclonal FITC-conjugated anti-tubulin antibody (1:100, Sigma, MO). After washing three times (5 min each) in PBS containing 1% Tween 20 and 0.01% Triton-X 100, oocytes were incubated with an appropriate secondary antibody for 1 hour at room temperature. After three times of washing, Propidium Iodide (PI) was used for chromosome staining. All immunostaining experiments were simultaneously conducted with negative control, in which the primary antibodies were not applied. Finally, oocytes were mounted on anti-fade medium (Vectashield, Burlingame, CA, USA), and examined under a Laser Scanning Confocal Microscope (LSM 710, Zeiss, Germany) equipped with the 40× objectives. ROI measurement was applied to quantify actin fluorescence of each oocyte images. Fluorescence intensity was randomly measured by plot profiling using ImageJ software (NIH, USA). At least 50 oocytes from three independent experiments were examined for each group.

### Chromosome spread

Chromosome spreading was performed as previously described [30]. Oocytes were exposed to Tyrode's buffer (pH 2.5) to remove zona pellucida, and then fixed in a drop of 1% paraformaldehyde with 0.15% TritonX-100 on a glass slide. Samples were labeled with CREST (1:500, Cat#:15–234, Antibodies Incorporated CA, USA) for 1 hour to detect kinetochores, and chromosomes were co-stained with Hoechst 33342. Laser scanning confocal microscope was used to examine the numbers of chromosome in oocytes. The presence of an abnormal number of chromosomes in oocyte (more or less than 20) is defined as aneuploidy.

### Statistical analysis

Data are presented as mean ± SD, unless otherwise indicated. Statistical comparisons were made with Student's *t* test and ANOVA when appropriate. *P* < 0.05 was considered to be significant.
